# Anakinra in pediatric acute fulminant myocarditis

**DOI:** 10.1186/s13613-022-01054-0

**Published:** 2022-08-26

**Authors:** Louise Maunier, Ramy Charbel, Virginie Lambert, Pierre Tissières, the CLOVIS study group, Simon Barreault, Mélissa Beggaz, Emre Belli, Ramy Charbel, Caroline Claude, Philippe Durand, Caroline Galeotti, Sébastien Hascoet, Virginie Lambert, Alice Maltret, Clémence Marais, Louise Maunier, Jordi Miatello, Luc Morin, Louise Othoniel, Bastien Provot, Adrien Schvartz, Pierre Tissieres, Isabelle Van Aershot, Joy Zogby

**Affiliations:** 1 Pediatric Intensive Care and Neonatal Medicine, AP-HP Paris Saclay University, Bicetre Hospital, 78, Rue du Général Leclerc, 94275 Le Kremlin-Bicêtre, France; 2 Paediatric Radiology, AP-HP Paris Saclay University, Bicetre Hospital, Le Kremlin-Bicêtre, France; 3 Paediatric Cardiology, Institut Mutualiste Montsouris, Paris, France; 4 Institute of Integrative Biology of the Cell, Paris Saclay University, CNRS, CEA, Gif sur Yvette, France; 5 FHU Sepsis Inserm/AP-HP/Paris Saclay University , Le Kremlin-Bicetre, France

**Keywords:** Myocarditis, Children, COVID-19, B19 parvovirus, PIMS–TS, MIS-C, Anakinra, IL-1 receptor antagonist

## Abstract

**Background:**

Acute fulminant myocarditis in children is associated with elevated mortality and morbidity with few advances in its medical management. Here we report a preliminary experience of children treated with IL-1 receptor antagonist associated with rapid myocardial function recovery.

**Methods:**

A retrospective case series of children admitted in the Pediatric Intensive Care Unit of the Bicêtre Hospital (AP–HP Paris Saclay University) between April 2020 and January 2022 with acute myocarditis. Children were treated with subcutaneous anakinra (an IL-1 receptor antagonist). Patients characteristics, and outcome are reported.

**Results:**

Of 10 children admitted with acute fulminant myocarditis, eight were treated with sub-cutaneous anakinra. Seven children had SARS-CoV-2 post-infective myocarditis associated with multisystem inflammatory syndrome in children (MIS-C) and one child Parvovirus B19 myocarditis. In all patients a rapid (< 24 h) improvement in myocardial function was observed with concomitant decrease in myocardial enzymes. All patients survived with full myocardial recovery.

**Conclusions:**

In this pilot study, use of IL-1 receptor antagonist in the initial treatment of acute fulminant myocarditis in children seems to be associated with rapid stabilization and recovery.

**Supplementary Information:**

The online version contains supplementary material available at 10.1186/s13613-022-01054-0.

## Introduction

Acute fulminant myocarditis is among the most severe condition in critically ill children for which supportive therapy remain the mainstay. However, aetiology-targeted treatment of myocarditis are missing and may prove to radically improve short and long-term prognosis [1]. Myocarditis has been defined as an inflammatory disease of the myocardium, established with histological, immunological criteria, with or without cardiac failure [2]. In children, myocarditis diagnosis is based on clinical presentation, biological markers (including Troponin T (TnT), NT-pro BNP), cardiac magnetic resonance imaging (MRI), and myocardial biopsy [1]. Myocarditis aetiologies are multiple, but post-infective mechanisms are well-identified as central trigger of the disease. Since the global pandemic of SARS-CoV-2 infection, post-infective myocarditis as part of the multi-inflammatory syndrome in children (MIS-C) was largely described in children and raised the possibility of immunomodulatory therapies with immunoglobulins (IVIg) and steroids [3, 4]. Carter et al*.,* in severe MIS-C with myocarditis, observed high serum concentrations of IL-1β, suggesting a potential role of IL-1 pathways in its pathogenesis [5]. In adults with acute myocarditis, activation of the inflammasome through the production of pro-inflammatory mediators such IL-1β, IL-18 is widely recognized [6]. The benefic effect of an IL-1 receptor antagonist in the treatment of acute myocarditis has been recently suggested [7, 8]. Anakinra, a recombinant human IL-1 receptor antagonist, neutralizes the biological activity of interleukin-1 alpha and beta (IL-1ß) by competitive inhibition of IL-1 binding to its type I receptor (IL-1RI). Thus we report our experience of eight children with acute fulminant myocarditis who received anakinra.

## Case series

**Fig. 1:**
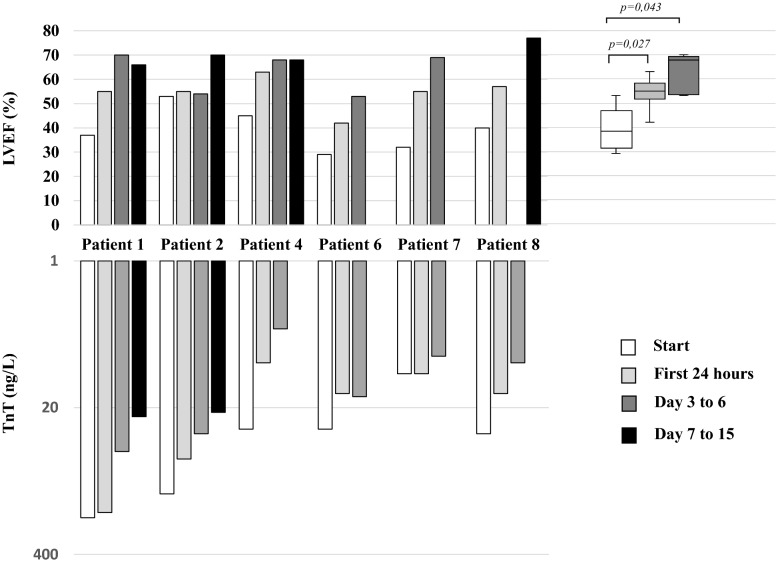
Left ventricle ejection fraction and serum troponin T following anakinra therapy. Values are expressed as LVEF (%) at days 0–2, days 3–6, and days 7–15 after Anakinra therapy. Wilcoxon sign rank test was used to test paired values. LVEF, Left Ventricle Ejection Fraction; TnT, Troponin T

Between April 2020 and January 2022, eight children with acute fulminant myocarditis were admitted to the Paediatric Intensive Care Unit (PICU) of the Bicêtre Hospital and were treated with sub-cutaneous Anakinra. Acute fulminant myocarditis was diagnosed using conventional criteria used in children: acute myocardial dysfunction identified by echocardiography in a previously healthy child without known cardiopathy associated with increased troponin and confirmed by cardiac MRI using revised Lake Louis criterion for diagnosis of myocarditis. Endomyocardial biopsy were not performed. During the same period, two additional children with acute fulminant myocarditis (Covid-19, Mediterranean fever) did not received anakinra. Patients characteristics are described in Table 1. The mean age was 7 years, ranging from 13 months to 14 years. Four of eight children were in cardiogenic shock and needed vasopressor drugs for a median of 1,5 days (1–4 days), one required central extracorporeal membrane oxygenation (ECMO). All but one patient had a Left Ventricular Ejection Fraction (LVEF) of less than 50% at admission in the PICU. The one patient who didn’t have left ventricular (LV) dysfunction was a 1-year-old boy with surgically corrected Tetralogy of Fallot with positive cardiac enzymes (TnT and NT-pro BNP) in the course of an active COVID-19 infection (positive SARS-CoV-2 PCR) associated with hematologically diagnosed macrophage activation syndrome. Two of the six children had ventricular arrhythmia, one evolving toward malignant ventricular fibrillation. Seven patients had post-infective myocarditis, six secondary to SARS-CoV-2 (positive IgG and negative PCR) and one following B19 parvovirus (positive IgG, negative IgM, negative PCR) infection. All six patients with MIS-C experienced a rapid (within 24 h following Anakinra first injection) LVEF improvement, decreased of TnT (Fig. 1) concomitantly to a decrease of blood C-reactive protein (Additional file 1: Figure S1).

**Table 1:** Patients characteristics

	Patient 1	Patient 2	Patient 3	Patient 4	Patient 5	Patient 6	Patient 7		Patient 8
Demographics									
Age (years)	14	13	1	4	1	9	10		5
Weight (kg)	62	86	8.2	17	7.9	38.9	38		24.5
Sex	Male	Female	Male	Male	Male	Female	Male		Male
Comorbidity	No	Obesity	Fallot tetralogy	No	No	No	No		No
Clinical condition									
Cardiogenic shock	Yes	Yes	No	No	Yes	Yes	No		No
Ventricular arrythmia	Yes	No	No	No	Yes	No	No		No
MAS	No	Yes	Yes	Yes	No	No	No		No
Cardiac evaluation at admission									
LVEF (%)	37	30	–	45	37	29	32		40
Subaortic VTI (cm)	13	18	–	14	8	12	13		14
LV dilatation	No	No	No	No	No	No	No		No
MI (grade)	Grade I	Grade I	–	Grade I	Grade III	Grade II	Grade II		Grade I
RV dysfunction	Yes	No	No	No	No	No	No		No
ECG	Fleeting ventricular arrhythmia	Normal	Normal	Normal	Vf bursts, st segment elevation	Normal	Normal		Normal
Laboratory findings at admission									
CRP (mg/l)	310	333	150	94	10	288	248		196
PCT (μg/l)	84.87	15.8	28.7	2.63	0.26	6.19	161.29		3.81
Wbcs (/mm^3^)	19,900	4870	16,690	11,230	10,260	17,090	5980		20,000
Lymphocytes (/mm^3^)	1350	370	1670	1680	2870	1590	950		1280
Neutrophils (/mm^3^)	17,270	4270	12,520	7640	6870	14,520	4800		18,260
Serum creatinine (μmol/l)	133	79	31	32	37	59	39		30
NT pro BNP (ng/l)	16,931	20,865	4269	2231	61,079	2591	7444		4105
PCR SARS-CoV-2 (blood)	Negative	Negative	Positive	Positive	Negative	Negative	Negative		Positive
IgG SARS-CoV-2	Positive	Positive	Negative	Positive	Negative	Positive	Positive		Negative
Other virus (blood)	No	No	No	No	PB19 (IgG + , IgM -, PCR -)	No	No		No
Treatment									
Immunotherapy	Anakinra + IVIg	Anakinra + IVIg + CS	Anakinra + IVIg+ CS	Anakinra + IVIg + CS	Anakinra + IVIg	Anakinra + CS	Anakinra + CS		Anakinra + CS
Reason for initiating anakinra	Poor hemodynamic tolerance of IVIg	Poor hemodynamic tolerance of IVIg	MAS (bone marrow analysis, high blood ferritin and triglycerides)	Poor hemodynamic tolerance of IVIg	Severity of clinical picture	Severity of clinical picture	Major inflammatory state		Major inflammatory state
Time to start anakinra after admission (days)	0	1	8	0	3	1	0		2
Total anakinra treatment time (days)	14	15	11	7	4	1	3		3
Anakinra dosage per day	200 mg	200 mg	6 mg/kg	4 mg/kg	4 mg/kg	2 mg/kg	4 mg/kg		4 mg/kg
Max VIS score	55	5	160	0	1275	20	0		0
Total inotropic/ vasopressor support duration (days)	1	1	4	0	4	2	0		0
Diuretic	Yes	Yes	No	No	Yes	Yes	No		No
Other cardiovascular therapy	No	No	Milrinone	No	Milrinone; amiodarone; levosimendan	No	No		No
Length of stay in ICU (days)	6	7	19	3	23	6	1		3
Length (days) of non-invasive ventilation	0	4	0	0	1	0	0		0
Length (days) of invasive ventilation	0	0	6	0	18	0	0		0

Vasoactive-Inotropic Score (VIS) = dopamine dose (μg/Kg/min) + dobutamine dose (μg/Kg/min) + 100 × epinephrine dose (μg/Kg/min) + 10 × milrinone dose (μg/Kg/min) + 10,000 × vasopressin dose (U/Kg/min) + 100 × norepinephrine dose (μg/Kg/min)

*BMI* body mass index, *MI* mitral insufficiency, *TTE* transthoracic echocardiogram, *LVEF* left venticular ejection fraction, *VTI* aortic velocity time integral, *ECG* electrocardiogram, *VF* ventricular fibrillation, *CRP* C-reactive protein, *PCT* procalcitonin, *WBCs* white blood cells, *MAS* Macrophage Activation Syndrome, *PB19* B19 Parvovirus, IVIg, immunoglobulins, *CS* corticosteroids

The 1-year-old boy with B19 parvovirus acute fulminant myocarditis demonstrated cardiogenic shock and malignant refractory ventricular fibrillation requiring central ECMO. Anakinra was administered on day 0 of ECMO and continued for 4 days in total. ECMO was successfully weaned after 10 days. 2 weeks following the initiation of anakinra, repeat transthoracic echocardiogram (TTE) demonstrated marked improvement in LVEF, from 13 to 55%, with a decrease in left end-diastolic ventricular diameter from 39.9 mm (Z-score 6.47) to 33 mm (Z-score 2.71). A cardiac MRI was performed 27 days following anakinra treatment and showed only a discrete basal and mid-lateral linear subepicardial enhancement. Four of the eight patients (3 MIS-C, 1 B19 parvovirus) had 5 months follow-up. All had normal LVEF and left ventricle longitudinal strain. No treated patients showed any recognized adverse events related to anakinra therapy.

## Discussion

Hereby, we report the use of anakinra in eight patients with acute fulminant myocarditis and its benefit on LV function recovery. The possible outcomes of myocarditis in children are sudden death, arrhythmias, myocardial infarcts and heart failure with dilated cardiomyopathy phenotype [1]. An analysis of the Pediatric Cardiomyopathy Registry (PCMR), including 369 children with myocarditis, found that 3 years after presentation approximately 7% of patients had died, 18% had undergone heart transplantation and 53% had normal left ventricular structure and systolic function [10]. In the current case series none of the patient died or were transplanted. All of them had achieved echocardiographic normalization and only two of six children had arrhythmia. Profibrotic cytokines including interleukin IL-1β play an essential role in tissue remodeling by stimulating fibroblast proliferation resulting in greater collagen synthesis and fibrosis [11]. In adults with acute myocarditis, formation of the inflammasome is a well-recognized pathogenic mechanism. Inflammasomes are a group of protein complexes which recognize a diverse set of inflammation-inducing stimuli, including Pathogen- and Damage-Associated Molecular Patterns, and control the production of several proinflammatory cytokines, such as IL-1β [6]. The use of early IL-1 receptor antagonist in patients identified with an inflammatory cardiomyopathy might further reduce progression to chronic dilated cardiomyopathy. In our series, all patient had high C-reactive protein suggestive of significant systemic inflammatory process. A randomized study in adults is currently underway comparing anakinra versus placebo for the treatment of acute myocarditis (ARAMIS, ClinicalTrials.gov Identifier: NCT03018834). Anakinra is a recognized therapy targeting the inflammasome in various systemic inflammatory disease, such as rheumatoid arthritis and juvenile polyarthritis [9]. In children, it is a therapy under investigation for Kawasaki disease that shows many clinical similarities with MIS-C [12, 13]. In our series, in most patients LVEF improvement was noted in the first hours following anakinra injection. The effect of other treatments such as corticosteroids cannot be excluded. Effect of IVIg is questionable. A systematic review of IVIg therapy in presumed viral myocarditis included only one randomized pediatric study of 86 children and found that IVIg did not significantly improve survival or left ventricular function [14]. Three patients did not hemodynamically tolerate large volume load secondary to IVIg infusion and treatment was interrupted. Altogether, currently neither corticosteroids nor IVIg ever showed such immediate recovery of contractile function during acute fulminant myocarditis. As for the treatment of MIS-C, use of IVIg, glucocorticoids, showed conflicting results. The US Overcoming Covid consortium [15] determined that initial treatment of MIS-C with immunoglobulin plus glucocorticoids was associated with a lower risk of cardiovascular dysfunction, requirement of adjunct therapies and vasopressors than with IVIg alone. In contrast, the international Best Available Treatment Study (BATS) consortium found no statistically significant difference for ventilation, inotropic support, death or for improvement on an ordinal clinical severity scale for any of the three treatments: immunoglobulin alone, a combination of immunoglobulin and glucocorticoids, or glucocorticoids alone [16]. This discrepency may be explained by different patient severity, different SARS-CoV-2 variants, but neither of these studies definitively answered the question about effective single or combination of immunomodulatory treatment including steroids, IVIg and biotherapies with monoclonal antibodies, such as anakinra and infliximab [17]. Although it is becoming increasingly clear that rapid immunomodulatory therapies can be lifesaving in patients with MIS-C, the underlying change in the therapeutic paradigm of acute fulminant myocarditis is changing and warrant randomized, controlled trials to evaluate the safety and efficacy of biotherapies in acute myocarditis.

In conclusion, our retrospective case series supports further investigation of the role for IL-1 receptor antagonist in the treatment of pediatric acute and fulminant myocarditis. All patients had rapid response to anakinra on contractile dysfunction and cardiac enzymes. Given its safety [18] and rapid onset of action, anakinra may have a place in the pediatric myocarditis treatment.

## Take-home message

Anakinra, an IL-1 receptor antagonist, is shown to be safe and may provide a novel approach to treat fulminant acute myocarditis in children.

## Supplementary Information

**Additional file 1: Figure S1.** Blood C-reactive protein response to anakinra.

## Data Availability

Upon request to corresponding author.
