# Supplementary material for: Anakinra in pediatric acute fulminant myocarditis
Source: Ann Intensive Care. 2022 Aug 26;12:80. doi: 10.1186/s13613-022-01054-0 (PMC9415255; doi:10.1186/s13613-022-01054-0)
Supplement: Supplementary file 1 — Additional file 1: Figure S1. Blood C-reactive protein response to anakinra. [file 13613_2022_1054_MOESM1_ESM.docx]

**Electronic Additional file 1 Material Figure S1: Blood C-reactive protein response to anakinra**


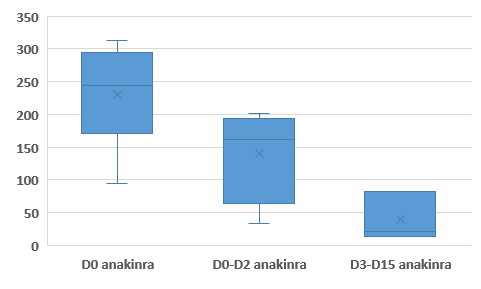


**Blood C-Reactive Protein (mg/L)**
